# Surgical Versus Non-Surgical Treatment of Patients with Myopathic Scoliosis: Clinical, Radiological and Functional Outcomes

**DOI:** 10.3390/children12111562

**Published:** 2025-11-17

**Authors:** Alexandra Satanovsky, Rana Hanna, Patrice L. Weiss, Amihai Rigbi, Josh E. Schroeder, Sharon Eylon

**Affiliations:** 1 Spine Unit, Orthopedic Complex, Hadassah Hebrew University Medical Center, Jerusalem 9112102, Israel; alexandras@hadassah.org.il (A.S.);; 2 Department of Internal Medicine, Rambam Health Care Campus, Haifa 31096, Israel; 3 The Helmsley Pediatric and Adolescent Rehabilitation Research Center, ALYN Hospital, Jerusalem 91090, Israel; tamarw@alyn.org; 4 Department of Occupational Therapy, University of Haifa, Haifa 3499003, Israel; 5 Department of Education, Beit Berl Academic College, Kfar Saba 4490500, Israel; arigbi@gmail.com; 6 Department of Pediatric Orthopedics, ALYN Pediatric and Adolescent Rehabilitation Center, 84 Shmariyahu Levin St., Jerusalem 91090, Israel; 7 Department of Orthopedics, Faculty of Medicine, Hebrew University of Jerusalem, Jerusalem 9112102, Israel

**Keywords:** myopathy, scoliosis, surgery, quality of life, outcomes

## Abstract

**Highlights:**

**What are the main findings?**

• In patients with myopathic scoliosis, both surgical and non-surgical groups showed similar worsening spinal deformity over time.
• Despite similar radiological outcomes, surgically treated patients reported higher overall quality of life and functional independence, particularly in self-image domains.

**What are the implications of the main findings?**

• Surgical correction may enhance subjective quality of life even when objective spinal improvement is limited, emphasizing the importance of patient-centered outcomes.
• Decision-making for scoliosis surgery in myopathic patients should carefully balance medical risks with potential long-term gains in function and well-being.

**Abstract:**

**Background/Objectives:** Myopathies are acquired or genetic muscle diseases causing weakness and wasting, leading to poor posture, impaired coordination, reduced daily function, and scoliosis. The objective of this ambispective study was to compare clinical, radiological, and functional outcomes of patients with myopathic scoliosis treated surgically or non-surgically. **Methods:** We identified 118 patients (55% male) with myopathy and scoliosis from ALYN Rehabilitation Hospital’s database (1990–2022). Mean age at first visit was 5.45 ± 5.27 years. Seventeen (14%) underwent scoliosis surgery; others were managed non-surgically. Due to unbalanced group sizes, comparative group analyses used propensity score matching (15 surgical, 30 non-surgical patients). Retrospective data included demographics, medical history, respiratory and mobility status, Cobb angle (CA), pelvic obliquity (PO), and surgical details when applicable. Prospective telephone interviews were conducted including SRS-22r Quality of Life (QoL), WHO-QoL, and Functional Independence Measure for Children (WeeFIM). **Results:** Longitudinal analysis showed significant or near-significant worsening over time in CA (*p* = 0.03) and PO (*p* = 0.08), regardless of treatment type but no significant difference between surgical and non-surgical groups in progression rates. Interview data, for 7 surgical and 6 non-surgical patients, revealed that surgical patients reported higher overall QoL, FIM, and SRS-22r self-image scores, but lower scores for SRS-22r pain, general function, and activity levels. **Conclusions:** Existing research and this study suggest that despite surgical risks, scoliosis correction in neuromuscular conditions generally leads to improved QoL. Findings highlight the complexity of surgical decision-making for myopathic scoliosis, where medical risks must be weighed against potential long-term functional and QoL outcomes.

## 1. Introduction

Myopathies refer to a large group of acquired or hereditary (genetic) diseases that share common musculoskeletal features. Weakness and wasting of the voluntary muscles especially affect the shoulder girdle, pelvis, neck and hip muscles, leading to poor head control, trunk imbalance, and discoordination and difficulty in activities of daily living (ADL) [1]. These patients have a high prevalence of neuromuscular scoliosis.

In contrast to the S-shaped curve typically found in idiopathic scoliosis, neuromuscular scoliotic deformities present as a long C-shaped curve in the coronal plane and include the sacrum. Initially, muscular support of the vertebrae is compromised, leading to positional collapsing; over time, as the spine grows, the scoliosis becomes structural [2]. Up to 90% of patients with myopathy have scoliosis, with rapid worsening of 16–24 degrees per year during the peak growth period [3,4]. The structural changes are associated with a reduction in lung function of 4–8% of vital capacity for every 10° Cobb angle (CA) [1,3,5,6]. The nature of myopathic disease is progressive [7], leading to the loss of independent mobility and need for a wheelchair [1,5]. This is known to accelerate the development of scoliosis [1,5].

There are three main treatment modalities of scoliosis for myopathic patients. The first is medications (e.g., steroid treatment for Duchenne disease) that slow the course of disease progression. Maintaining mobility results in a reduction in the progression of scoliosis and the age of its development [1,3]. The second treatment modality is Thoraco-Lumbo-Sacral orthosis (TLSO), which has been demonstrated to be effective in adolescent idiopathic scoliosis (AIS) [4]. This treatment has questionable effectiveness for myopathic patients with scoliosis [1,3,5,6]. Surgery for scoliosis is the third and commonly accepted treatment for myopathic patients. At this stage of myopathy, patients are usually wheelchair bound, and the purpose of surgery is to improve sitting ability and prevent further deterioration of scoliosis and respiratory function [8].

The decision to perform surgery is complex. On the one hand, there is a desire to correct the spine and prevent the expected deterioration despite the risk of potential surgical complications. On the other hand, delaying surgery when there is a clear indication for its need (CA > 20 degrees) [3] results in the operation being performed when there is poor respiratory reserve, and thus possible greater postoperative complications. Moreover, the optimal timing of surgery remains without a clear answer in the literature [1,3,9].

The general rate of surgical complications described in myopathic patients is higher than AIS [7,10,11]. In their review of 2154 neuromuscular scoliosis fusion cases, Rumalla et al. [12] reported an overall complication rate of 40.1%, whereas others reported rates as high as 63% [11,13]. Additionally, an increasing number of operations has been reported [8,14], attributed to rise in new techniques [15] and growth-friendly procedures [16]. This naturally results in a rise in complications [17].

Similarities in curve presentation across a wide range of neuromuscular diagnoses (e.g., CP, SMA) lead to converging surgical indications and operative strategies, an issue also reflected in the literature [7,16]. Furthermore, most studies lack an appropriate comparator group, as they examined differences in surgical outcomes among patients with varying diagnoses rather than comparing the effectiveness of surgical versus non-surgical treatment within the same diagnosis [3,7,17]. Hence, drawing conclusions regarding surgical decision-making is complex [7].

While surgery of myopathic patients demands meticulous preparation and intraoperative adaptations in anesthesia due to unique metabolic, respiratory, and hematological factors [10,18], the added value of surgical treatment (e.g., to improve respiratory outcomes) is not clear in the literature [1,9]. Surgery aims to address the patient’s sitting position, allowing for adequate trunk position and reducing the frequency of pressure sores. This enhances participation in everyday life. In a study of patients with myopathy and scoliosis who were treated non-surgically, we did not see a direct relationship between the deterioration of the scoliosis and increases in respiratory distress [19]. More importantly, the results showed that they were satisfied with their quality of life, although they reported limited independence and were least satisfied with functional activity (whether or not this was related to the deformity or the myopathy itself) [19].

In a review of the recent literature, most studies focused on radiographic rather than patient-centered outcomes [18]; none indicated an improvement in quality of life, an extension of life, nor a change in the natural course of the disease as a result of surgical treatment [5]. Indeed, understanding patients’ experiences including barriers and facilitators of participation is suggested to improve clinical trial management [20]. Therefore, it is difficult to estimate the effect of surgery on the course of the disease in comparison to the natural history of patients who did not undergo surgery.

The aim of the current study was to compare two groups of patients with myopathies and scoliosis—those who had surgery compared to those who did not, in terms of their scoliotic deformity progression, respiratory status, mobility, pain, daily functioning, and overall quality of life. A preliminary version of the results was presented at the 2023 Global Spine Congress [21].

## 2. Materials and Methods

### 2.1. Study Design

**Figure 1:**
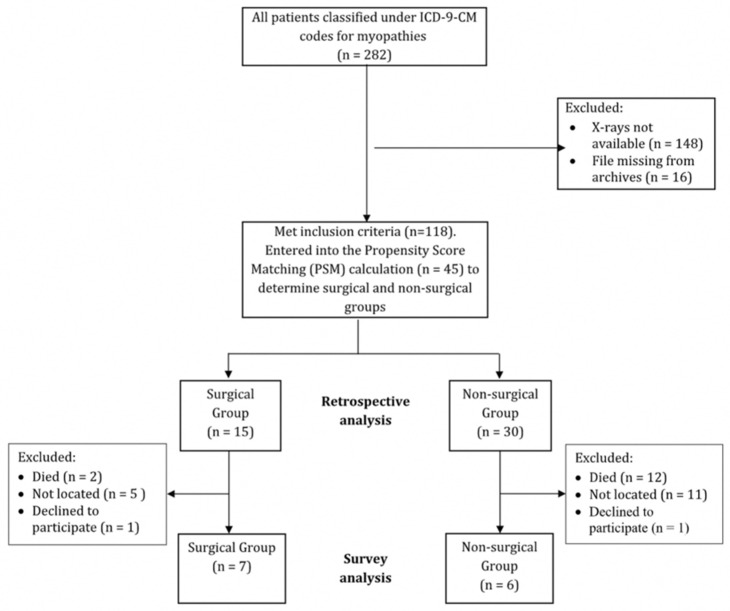
Flow diagram showing the recruitment and analysis stages for the retrospective and survey stages of the study.

The study employed an ambispective design to compare two groups of patients. Figure 1 illustrates the recruitment process, which involved extracting retrospective data from the hospital database (1990–2022) and archived files to identify patients with myopathy and scoliosis who underwent surgery. These individuals were then compared with a control group of non-surgical patients. To supplement the historical data, additional information was collected through a telephone survey conducted between September and October 2022. This survey included validated measures such as the Scoliosis Research Society-22R instrument (SRS-22r) Quality of Life (QoL) questionnaire, the World Health Organization (WHO) Quality of Life (WHO-QoL) assessment, and the Functional Independence Measure for Children (WeeFIM, Uniform Data System for Medical Rehabilitation, Buffalo, New York, USA). The study was approved by the ALYN Hospital Ethics Committee in accordance with the World Medical Association Declaration of Helsinki (approval number 054-22).

### 2.2. Patients and Research Team

Retrospective data from the hospital database (1990–2022) were included if patients were classified under the International Classification of Diseases, Ninth Revision, Clinical Modification (ICD-9-CM) codes for myopathies [22]. Each of the files was reviewed manually by the research team to confirm the diagnosis and its coding, updated to supply telephone interviews and questionnaires collected September–October 2022, including WHOQOL-BREF and SRS-22r Quality of Life (QoL) and Functional Independence Measure for Children (WeeFIM). Informed consent was obtained from participants of the telephone survey.

The team included one pediatric orthopedic rehabilitation specialist, two pediatric spine orthopedic surgeons, one medical student, and two research methodology experts. One of the pediatric spine orthopedic surgeons performed all the radiological measurements. Treatment protocols have been consistent over the years.

### 2.3. Context

The study was conducted at ALYN Hospital, a Pediatric and Adolescent Rehabilitation Center that cares for children with complex physical and medical conditions, offering a comprehensive range of rehabilitation services aimed at supporting healthy, independent adulthood. The hospital has 120 inpatient beds, with twice that number of children treated as outpatients. As a referral center for rehabilitation, treatment plans were established by external clinicians including the selection of patients for surgical correction.

### 2.4. Instruments

Demographic and medical questionnaire: This section included general demographic data, along with medical history, respiratory condition, and mobility status.

Cobb angle: The CA is a key measure used to assess the severity and progression of scoliosis. It is determined by drawing lines parallel to the upper and lower vertebrae of the structural curves in the spine and erecting perpendiculars from these lines, which intersect to form the CA [23]. The CA, typically measured on a standing coronal radiograph of the full spine, remains the gold standard for scoliosis evaluation [24].

Pelvic Obliquity: PO refers to the misalignment of the pelvis, marked by its tilt in the coronal plane. It was measured using the Maloney Method, where a line is drawn across the iliac crests, a perpendicular line is added, and another line connects T1 and S1. The angle at which the second and third lines intersect defines PO. This method shows high reliability (ICC = 0.96) [25,26].

World Health Organization Quality of Life (WHOQOL)-BREF: The WHOQOL-BREF is a 26-item questionnaire that assesses self-reported quality of life. It demonstrates strong internal consistency (Cronbach α = 0.92), good test–retest reliability (r = 0.66–0.72), and good concurrent validity [27].

Scoliosis Research Society-22 revised (SRS-22r Quality of Life): The SRS-22r evaluates health-related quality of life in adolescents with idiopathic scoliosis. It shows good interrater reliability (ICC = 0.61–0.9) and good internal consistency for most domains (Cronbach α = 0.71–0.85), although the activity/function and satisfaction with management domains have slightly lower internal consistency (Cronbach α = 0.44–0.58) [28,29].

Functional Independence Measure for Children (WeeFIM): The WeeFIM is an 18-item tool used to assess disability in children. It demonstrates strong reliability (ICC = 0.8–0.99) [30,31] and high concurrent validity in self-care, mobility, and communication/social function domains (r > 0.88) [32].

### 2.5. Data Retrieval and Analysis

Descriptive statistics were used to characterize the patients’ demographics, medical data, functional status, and quality of life (QoL). Categorical and ordinal variables were summarized using frequencies and percentages, while continuous variables were described using means, medians, and standard deviations. Inferential statistics were employed to compare outcomes between patients receiving surgical versus non-surgical treatment and examine associations with medical sequelae and changes in QoL, mobility, social functioning, and satisfaction. Due to the substantial imbalance between groups: 101 non-surgical patients (86%) and 17 surgical patients (14%), propensity score matching (PSM) was applied to support comparative analysis. This quasi-experimental method enables the creation of covariate-balanced groups by matching similar cases (surgical group in the context current paper) and controls (non- surgical group), thereby minimizing potential confounding effects of covariates.

Optimal pair matching was selected for the PSM procedure as it minimizes the total distance in propensity scores across matched pairs and improves the overall balance and reliability of the matched sample [33]. PSM was performed using the matchit package in R software (version 4.3.1), with matching quality assessed and visualized using the cobalt package. Matching was based on a 2:1 ratio (two non-surgical patients per one surgical patient), using three variables: age at first visit, CA at first visit, and diagnosis. Following matching, no significant differences were found between the surgical and non-surgical groups on these variables (see Section 3).

To assess group differences in the matching variables, the Wilcoxon rank-sum test (with continuity correction) and the Fisher exact test were applied. Given the small sample size and non-normal distribution of the dependent variables, a nonparametric, distribution-free approach was adopted using the *nparLD* package in R software. This method enables the nonparametric analysis of longitudinal data in factorial designs [34], and the data were analyzed within a between-subjects (group) by within-subjects (time) framework. All statistical analyses were conducted using SPSS version 26 and R version 4.3.1. A two-tailed *p*-value ≤ 0.05 was considered statistically significant.

## 3. Results

The initial database search identified 282 potential cases with ICD-9 codes for myopathy and scoliosis. Following verification of diagnosis and availability of radiographic data, 118 patients were included in the retrospective analysis (17 surgical, 101 non-surgical). For comparative analysis, propensity score matching produced a balanced subset of 15 surgical and 30 non-surgical cases (2 surgical patients were excluded due to incomplete baseline data required for matching). As mentioned, the matching was based on three variables: age at first visit to ALYN Hospital, CA at baseline (i.e., first visit), and diagnosis. Table 1 presents the results of the matching procedure, indicating no significant differences between the non-surgical and the surgical groups on these variables.

**Figure 2:**
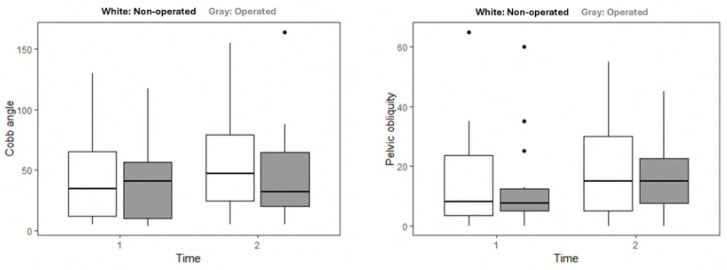
The Cobb angle (**left**) and pelvic obliquity (**right**) at first (1) and last (2) clinical visits of non-surgical (white) and surgical (grey) patients. Horizontal lines represent the median and the whiskers represent 25% and 75% percentiles.

Figure 2 illustrates the progression of CA and PO in surgical and matched non-surgical patients over time. As demonstrated in Figure 2 (left) and detailed in Table 2, the CA increased in the non-surgical group whereas it decreased in the surgical group. In terms of PO (Figure 2, right), a rise in the median value was observed in both groups over time. Notably, one patient in the surgical group exhibited an extreme increase in PO from 5 to 45 degrees between visits. Given the small sample size, this individual case may have disproportionately influenced the group’s overall trend and should be interpreted with caution. Omitting this patient from the analysis would have resulted in a median PO increase from 10 degrees at the first visit to 14 degrees at the last visit. However, this patient remained in the subsequent analyses in order to avoid selective data omission.

Due to the small group size, a nonparametric analysis, based on ranking for longitudinal data. was performed to test the effect of time and group type and the interaction between them on CA and PO. The inferential analysis revealed a significant, or close to significant main effect for time for both measures (CA: Wald_(1)_ = 5.71, *p* = 0.03; PO: Wald_(1)_ = 3.01, *p* = 0.08), indicating that overall, the CA and PO worsened over time (despite the decrement of the median CA in the surgical group). The main effect for group (surgical versus the non-surgical) was non-significant (CA: Wald_(1)_ = 0.32, *p* = 0.57; PO: Wald_(1)_ = 0.01, *p* = 0.90) as was the interaction between group and time (CA: Wald_(1)_ = 0.58, *p* = 0.44; PO: Wald_(1)_ = 0.00, *p* = 0.94).

The questionnaire data following the PSA procedure included only seven surgical patients and six non-surgical patients; out of the remaining 32 surgical and non-surgical patients, 14 died, 16 could not be reached by phone, and 2 declined to participate (Figure 1).

As shown in Table 3, the surgical patients reported higher QoL, FIM, and SRS-22r general self-image scores, and lower SRS-22r pain, general-function, and function activity scores; only the QoL difference was statistically significant (Mann–Whitney test: z = −2.29, *p* = 0.02).

Comparison of the SRS-22r scores for the seven surgical patients revealed no meaningful differences between pre- and post-surgical reports. The median (interquartile range) for the SRS General Self-Image decreased from 4.66 (1.00) before surgery to 3.66 (1.00) after surgery, while the SRS General Function increased from 3.66 (0.67) pre-surgery to 4.00 (4.00) post-surgery. However, these changes were not statistically significant.

## 4. Discussion

Patients with myopathy represent a rare group reaching adulthood while facing significant daily challenges. Our previous study on their natural history [19] showed that respiratory and mobility changes may occur independently of scoliosis progression, and that many maintain an active lifestyle even without scoliosis surgery [19]. Surgical decisions depend on deformity severity, age, diagnosis, and comorbidities [13,35], while addressing issues such as sitting balance, pain, respiratory complications, and quality of life [7,13,15,36,37]. Despite careful preparation, risks remain high, with 1% in-hospital mortality [13,38,39] and 40–63% major complications [11,12,13]. Unlike prior literature comparing myopathy to cerebral palsy [40], the current study directly contrasts myopathic patients with scoliosis who have either had surgery or who have not.

Looking at the radiological findings, the only significant parameter concerning progression of scoliosis and PO of both the surgical and non-surgical groups was time. No statistically significant difference was found between the surgical and non-surgical groups in the severity of the final CA, as shown in Table 2. The final CA of surgical patients was similar to the first measurements in the non-surgical group. While the non-surgical patients’ scoliosis worsened from 34.5 to 47 degrees, in the surgical group, the measurements improved from 41 to 32 degrees. Sitting ability, as represented in PO, worsened over time in the non-surgical group.

Regarding the questionnaire responses, the patients who had surgery reported significantly increased scores for QoL (WHOQOL-BREF). Results from the other questionnaire outcome measures did not reach statistical significance, although the FIM and SRS-22r general self-image scores were higher for the surgery group; they also experienced decreased pain and a lower self-image in the SRS-22r. These results are supported by previous studies that highlight the importance of taking into consideration the patient’s goals, preferences, and expectations as well as their reported post-surgical QoL [7,41,42,43].

There are several limitations to this study. First, the retrospective nature of it may lead to variation in information quality, with variations over the years. This may have only had a limited impact on data quality as the clinical personnel have remained stable over time. Second, patients with missing radiographic data were excluded, leading to possible bias in the final results. Third, a key limitation of this study was the small number of participants who completed the prospective assessments (*n* = 13). This sample size restricts statistical power and increases the likelihood of type II errors, meaning that true group differences may not have been detected. The rarity of myopathic scoliosis, compounded by mortality and loss to follow-up in this long-term (up to 32 years) cohort, contributed to the reduced number of participants. Although we employed nonparametric analyses and PSM to enhance robustness and reduce bias, the results should be interpreted with caution. The findings are best viewed as exploratory and as a foundation for future, larger-scale, multicentric investigations aimed at validating these trends.

Fourth, although PSM improved comparability between the surgical and non-surgical cohorts by controlling for age, diagnosis, and curve severity, the influence of additional unmeasured confounders cannot be excluded. Differences in baseline function, degree of sitting balance, or preoperative pain may have affected both the indication for surgery and postoperative adaptation. Likewise, variations in family support or psychosocial context could have influenced quality-of-life outcomes independently of surgical status. These factors highlight the inherent limitations of retrospective designs and the importance of future prospective studies incorporating comprehensive functional and contextual data.

Last, because of the rare nature of these disorders, the overall number of patients is still small relative to other studies. To the best of our knowledge, this is the largest report, to date, of patients with myopathic scoliosis comparing patients who underwent surgery to those who did not. Increasing the sample size via a multicentric study is recommended. Finally, although the number of patients reporting pre- and post-surgery SRS-22r scores was small, presenting their responses is valuable since they highlight the patients’ perspectives.

## 5. Summary and Conclusions

The results of this study highlight the complex dilemma faced by both families and clinicians when deciding whether to proceed with scoliosis surgery for patients with myopathy. For some children, the decision is guided primarily by medical and physical considerations; for instance, while respiratory distress may make surgery risky, timely scoliotic correction could prevent further respiratory decline. At the same time, families often express concerns about the potential complications of surgery and question its long-term benefits, particularly regarding respiratory function, mobility, pain relief, and quality of life. Although these decisions must be approached with caution due to the lack of definitive evidence favoring surgery as the superior option, existing research suggests that, despite the significant risks, QOL tends to improve for patients undergoing surgical scoliosis correction in neuromuscular conditions, as supported by the present study and others [7]. These considerations must be carefully communicated by the treating team to enable families to make informed decisions [20].

Although the number of participants completing the prospective phase was limited, the ambispective design of this study remains meaningful. It enabled the integration of long-term, systematically collected retrospective clinical and radiological data with contemporary, patient-reported outcomes, an approach seldom feasible in this rare population. The large retrospective component provided longitudinal context and objective measures of disease progression, while the smaller prospective subset offered insights into quality of life and functional independence. Together, these data illustrate the continuum between the medical and lived experiences of patients with myopathic scoliosis. There is a need for further study, preferably multicentric, due to the rarity of the diagnoses.

## Figures and Tables

**Table 1:** Results of the propensity score matching used to support comparative analysis of the non-surgical and the surgical groups based on the participants’ age and Cobb angle at their first clinical visit, and diagnosis. * Two participants were omitted from the PSA procedure due to the listwise deletion. Md.—median, IQR = interquartile range.

Matching Variable	Non-Surgical (*n* = 30)	Surgical (*n* = 15 *)	Significance
Age at first visit (Md., IQR)	6.00, 13	4.90, 9.9	Wilcoxon rank sum test: *p* = 0.22
Cobb angle at baseline (Md., IQR)	34.50, 56	41, 51	Wilcoxon rank sum test: *p* = 0.79
	Number (%)	Number (%)	
Diagnosis			Fisher exact test: *p* = 0.62
Myopathy, unspecified	10 (33)	3 (20)	
Congenital myopathy—fiber type disproportion	4 (13)	5 (33)	
Congenital muscular dystrophy	12 (40)	5 (33)	
Congenital myopathy—nemaline	2 (7)	1 (7)	
Spinocerebellar disease	2 (7)	1 (7)	

**Table 2:** Medians and interquartile ranges for Cobb angle and pelvic obliquity at first and last clinical visits of the non-surgical and the surgical groups. Md.—median, IQR = interquartile range.

	Non-Surgical		Surgical	
	First Visit	Last Visit	First Visit	Last Visit
Cobb Angle Md. (IQR)	34.5 (53.2)	47 (54.7)	41 (46.5)	32 (44.5)
Pelvic Obliquity Md. (IQR)	8 (20)	15 (25)	7.5 (7.2)	15 (15)

**Table 3:** Medians and interquartile ranges for Quality of Life, Functional Independence Measure and SRS-22r at first and last clinical visits of the non-surgical and the surgical groups. * Difference was statistically significant. Md.—median, IQR = interquartile range. QoL = Quality of Life, WeeFIM = Functional Independence Measure for Children, SRS = Scoliosis Research Society-22R instrument.

Measure	Non-Surgical Patients Md. (IQR) (*n* = 6)	Surgical Patients Md. (IQR) (*n* = 7)
QoL *	3.40 (0.88)	4.15 (0.56)
WeeFIM	3.47 (1.15)	4.28 (3.88)
SRS general self-image	4.50 (1.17)	4.66 (1.00)
SRS pain	4.71 (0.93)	4.00 (0.86)
SRS general function	4.33 (0.75)	3.67 (0.67)
SRS Function activity	3.67 (0.33)	2.33 (1.00)

## Data Availability

The original contributions presented in the study are included in the article, further inquiries can be directed to the corresponding author.
